# Co-existence of a pandemic (SARS-CoV-2) and an epidemic (Dengue virus) at some focal points in Southeast Asia: Pathogenic importance, preparedness, and strategy of tackling

**DOI:** 10.1016/j.lansea.2022.100046

**Published:** 2022-07-20

**Authors:** Sakirul Khan, Sheikh Mohammad Fazle Akbar, Akira Nishizono

**Affiliations:** a Department of Microbiology, Faculty of Medicine, Oita University, Yufu, Oita 879-5593, Japan; b Department of Gastroenterology and Metabology, Ehime University Graduate School of Medicine, Toon, Ehime 791-0295, Japan; c Clinical Research Organization, Dhaka 1213, Bangladesh; d Research Center for Global and Local Infectious Diseases, Faculty of Medicine, Oita University, Yufu, Oita 879-5593, Japan

The coronavirus disease 2019 (COVID-19) pandemic, caused by severe acute respiratory syndrome coronavirus 2 (SARS-CoV-2), has now entered its 3rd year. Other infectious diseases with similar symptoms (dengue infection) usually prevail at different intensities in Southeast Asian and Latin American countries. A recent study published in *The Lancet Infectious Diseases* reported that COVID-19-related changes in social activities decreased the incidence of dengue during the first year of the pandemic (in 2020).^1^ Here, we provide data to support that the observed reduction in the incidence of dengue in 2020 may not be committed epidemiological behavior of dengue in 2021 in some South Asian countries (Figure 1; Suppl. Figure 1). Using the official database of (WHO/Government) and reference reporting system,^2^, ^3^, ^4^, ^5^, ^6^, ^7^ we have shown that a sporadic surge of dengue infection was recorded in some Southeast Asian countries in the 2nd year of the COVID-19 pandemic (2021) using Microsoft® Excel and SAS (version 9.4 (Cary, NC, USA).

**Figure 1:**
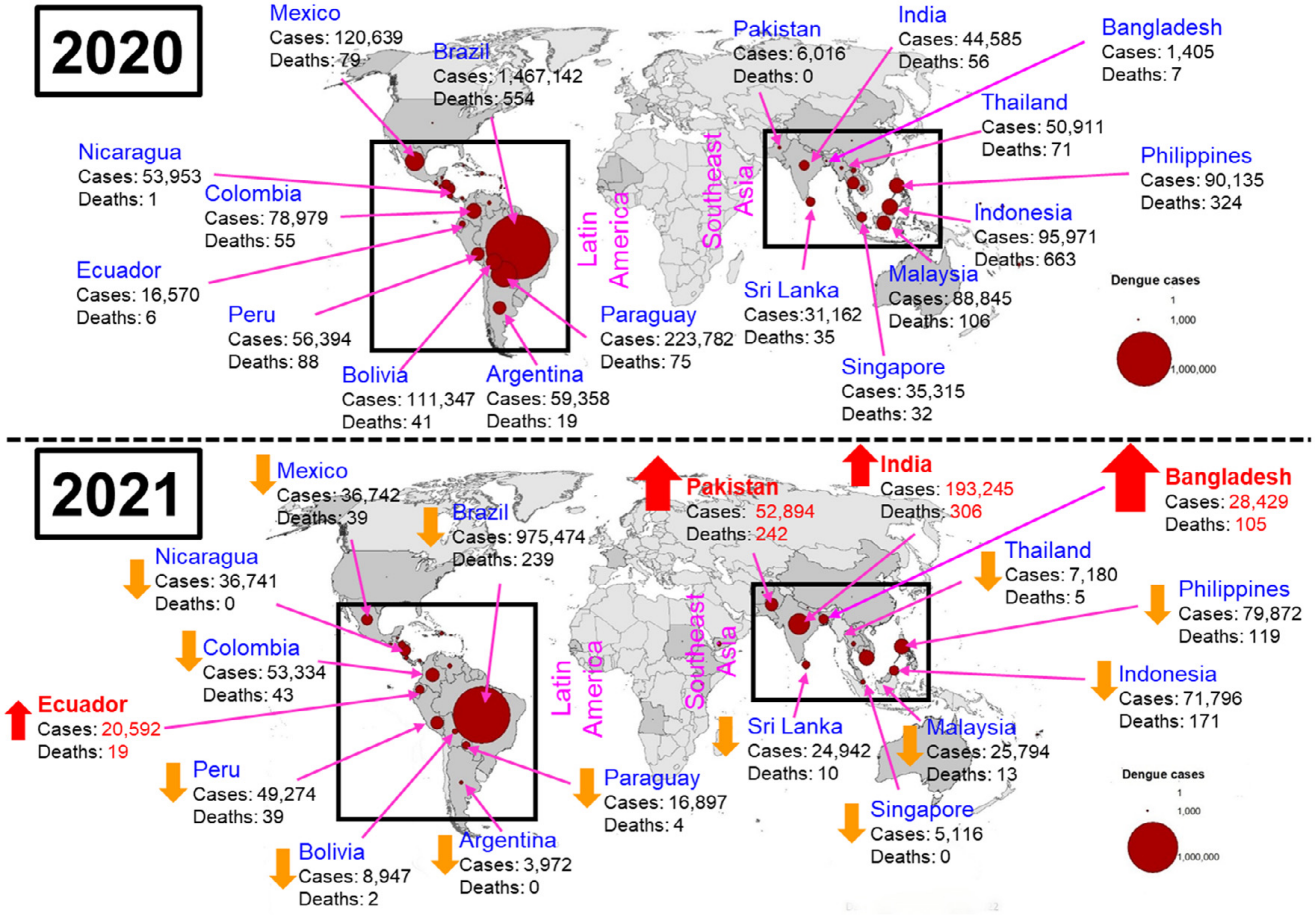
Geographical distribution of dengue cases during the coronavirus disease 2019 (COVID-19) pandemic period (2020∼2021). The upper panel represents dengue cases and deaths in Southeast Asian and Latin American countries during the first year of the COVID-19 pandemic (in 2020). The lower panel represents dengue cases and deaths in Southeast Asian and Latin American countries during the second year of the COVID-19 pandemic (in 2021). Upward arrows indicate an increase of dengue cases and deaths, and downward arrows indicate a decrease of dengue cases and deaths.

Overall, the dengue cases declined by approximately 36% (2,795,822 vs. 1,788,229) in 2021 compared to 2020 across the study area in Southeast Asia and Latin America. However, 4 of the 22 countries (Ecuador, Bangladesh, Pakistan, and India) recorded an increased number of dengue cases in the second year of the COVID-19 pandemic (Figure 1; Suppl. Figure 1). India reported an increase in dengue incidence of over 3-fold (44,585 vs. 193,245 cases), while Pakistan showed an increase of greater than 7-fold (6016 vs. 52,894 cases). In contrast, Bangladesh reported an increase of more than 19-fold (1405 vs. 28,429) in 2021 compared with those reported in 2020. However, the article in *The Lancet Infectious Diseases*^1^ failed to include these three South Asian countries in their analysis. The increased incidence of dengue cases was also associated with increased dengue-related deaths in 2021 compared with those for 2020 (Bangladesh: 7 vs. 105; Pakistan: 0 vs. 242; India: 56 vs. 306). However, this pattern was not observed in any other country (Figure 1, Suppl. Figure 1). When we compared the average changes in dengue cases for each region between 2020 and 2021, countries in Southeast Asia reported a higher incidence than Latin America in 2021 (261% vs. −156%; *p*<0.05). Moreover, in contrast to Latin America, a strong positive correlation between dengue cases/million population and COVID-19 cases/million population has been observed in Southeast Asian countries (r = 0.10 vs. r = 0.83).

Although COVID-19-related social measures are thought to reduce dengue infections during the pandemic,^1^ the increased incidence in South Asian countries is somewhat surprising because these three countries applied relatively strong COVID-related measures in 2021 after facing massive outbreaks of the delta variant of SARS-CoV-2. In addition, the clinical characteristics of patients infected with dengue in 2021 presented with atypical symptoms, quickly progressing to severe illnesses, and endowed with neurological and cardiac complications, especially in children, in contrast to traditional dengue-related symptoms. These indicate that the clinical severity of dengue cases in South Asian countries in 2021 may be partly attributable to co-infection with SARS-CoV-2 and dengue virus.^8^ This may also be due to a second or third infection with dengue, which is not uncommon in these countries. Usually, dengue re-infection may present as a more severe disease due to antibody-dependent enhancement (ADE).^9^ Evidence has also suggested that SARS-CoV-2 is capable of inducing ADE.^10^ Thus, patients with a previous infection of dengue virus with present SARS-CoV-2 infection or co-infection of these two viruses may lead to abrupt development of severe complications due to possible additive effects of the ADE of these two viruses.^8^ We are not sure at this moment if either virus has a role in inducing a deadly infection at the cellular and molecular levels. This knowledge should be kept in mind to manage both dengue and COVID-19 during the ongoing pandemic, as the world continues to face increased daily reports of dengue and COVID-19 cases with almost no approach to gain insights into the underlying mechanisms to prevent the worst outcomes from these two intractable diseases.

In 2022, the world will face scattered relaxation of COVID-19-related restrictions, even though the surge of the highly transmissible Omicron variant of SARS-CoV-2. This may allow for the spread of severe dengue infections across countries. As of June 22th, 2022, when the article was compiled, the incidence and prevalence of dengue are tremendously rising in many Southeast Asian and Latin American countries.^2^, ^3^, ^4^, ^5^, ^6^ For example, Brazil and Peru have already exceeded the number of dengue cases reported in 2021, and Nicaragua and Colombia show similar increasing patterns.^2^ In Southeast Asia, Singapore, the Philippines, Indonesia, and Thailand have already reported a significant number of dengue cases even though the dengue season has just begun in this region.^4^ The prediction analysis revealed that Bangladesh, India, and Nepal might account for more dengue cases in 2022 (article on preparation). Considering the severe condition of the double punch of a pandemic (COVID-19) and an epidemic (dengue) with similar and overlapping clinical symptoms, it is essential to develop a comprehensive and evidence-based scientific program to control the spread of dengue infections during the ongoing pandemic. More insights about epidemiology, pathogenesis, and management should be immediately planned with an eye on the strengths and weaknesses of healthcare delivery services in developing and resource-constrained countries with a notable dengue prevalence.

## Contributors

S.K. planned and compiled the manuscript. S.M.F.A. and A.N. provided support with the data, literature searches, and manuscript editing. All the authors have discussed and approved the final version of the manuscript.

## Data sharing statement

The corresponding author had full access to all data in the study and had final responsibility for the decision to submit for publication. The data that support the findings of this study are available on request from the corresponding author [S.K.].

## Declaration of interests

The authors have no commercial or other associations that might pose conflicts of interest.

## Appendix Supplementary materials

Image, application 1
