# Supplementary material for: Co-existence of a pandemic (SARS-CoV-2) and an epidemic (Dengue virus) at some focal points in Southeast Asia: Pathogenic importance, preparedness, and strategy of tackling
Source: Lancet Reg Health Southeast Asia. 2022 Jul 20;4:100046. doi: 10.1016/j.lansea.2022.100046 (PMC9296506; doi:10.1016/j.lansea.2022.100046)
Supplement: Supplementary file 1 [file mmc1.docx]

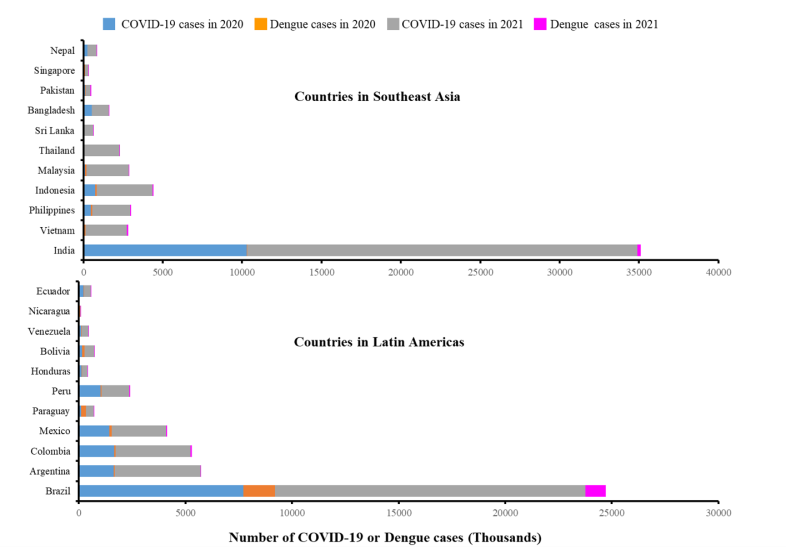


**Suppl. Figure Legend**

**Suppl. Figure 1. Serial observation of the pandemic coronavirus disease 2019 (COVID-19) and dengue cases during the pandemic.**  Periodic compilation of COVID-19 and dengue cases in Southeast Asian (upper panel) and Latin American (lower panel) countries during the pandemic period (2020–2021).
